# The multivariate physical activity signature associated with metabolic health in children

**DOI:** 10.1186/s12966-018-0707-z

**Published:** 2018-08-15

**Authors:** Eivind Aadland, Olav Martin Kvalheim, Sigmund Alfred Anderssen, Geir Kåre Resaland, Lars Bo Andersen

**Affiliations:** 1 Department of Sport, Food and Natural Sciences, Faculty of Education, Arts and Sports, Western Norway University of Applied Sciences, Campus Sogndal, Box 133, 6851 Sogndal, Norway; 2 Department of Chemistry, University of Bergen, Box 7800, 5020 Bergen, Norway; 3 Department of Sports Medicine, Norwegian School of Sport Sciences, Box 4014 Ullevål Stadion, 0806 Oslo, Norway; 4 Center for Health Research, Førde Central Hospital, Box 1000, 6807 Førde, Norway

**Keywords:** Multivariate pattern analysis, Metabolic risk factors, Pediatric, Childhood, Accelerometer, Intensity

## Abstract

**Background:**

Physical activity is a cornerstone for promoting good metabolic health in children, but it is heavily debated which intensities (including sedentary time) are most influential. A fundamental limitation to current evidence for this relationship is the reliance on analytic approaches that cannot handle collinear variables. The aim of the present study was to determine the physical activity signature related to metabolic health in children, by investigating the association pattern for the whole spectrum of physical activity intensities using multivariate pattern analysis.

**Methods:**

We used a sample of 841 children (age 10.2 ± 0.3 years; BMI 18.0 ± 3.0; 50% boys) from the Active Smarter Kids study, who provided valid data on accelerometry (ActiGraph GT3X+) and several indices of metabolic health (aerobic fitness, abdominal fatness, insulin sensitivity, lipid metabolism, blood pressure) that were used to create a composite metabolic health score. We created 16 physical activity variables covering the whole intensity spectrum (from 0–100 to ≥ 8000 counts per minute) and used multivariate pattern analysis to analyze the data.

**Results:**

Physical activity intensities in the vigorous range (5000–7000 counts per minute) were most strongly associated with metabolic health. Moderate intensity physical activity was weakly related to health, and sedentary time and light physical activity were not related to health.

**Conclusions:**

This study is the first to determine the multivariate physical activity signature related to metabolic health in children across the whole intensity spectrum. This novel approach shows that vigorous physical activity is strongest related to metabolic health. We recommend future studies adapt a multivariate analytic approach to further develop the field of physical activity epidemiology.

**Trial registration:**

The study was registered in Clinicaltrials.gov (www.clinicaltrials.gov) 7th of April 2014 with identification number NCT02132494.

## Background

Physical activity (PA) is a cornerstone for promoting good metabolic health in children [1, 2]. Specifically, moderate-to-vigorous PA (MVPA) has consistently been associated with single risk factors as well as with composite measures of metabolic health in childhood [1–4]. Additionally, sedentary time (SED), defined as time spent sitting or reclined with an energy consumption minimally above resting values (< 1.5 metabolic equivalents) [5], has received great attention for possibly being detrimental to child health beyond overall PA or MVPA [6–8]. However, the evidence for an influence of SED beyond MVPA on metabolic health in children is weak [3, 9].

The majority of pediatric studies investigating relationships between PA and metabolic health have been limited to investigating associations for MVPA and SED [1]. This narrow focus causes a substantial loss of information from accelerometry. Moreover, it ignores the possible influence of light PA (LPA), moderate PA (MPA) and vigorous PA (VPA), and increase susceptibility of residual confounding for analyzed variables [8]. Thus, associations with metabolic health for the whole PA intensity spectrum should be addressed [2, 8]. Given the relationships between VPA, aerobic fitness, and cardiovascular risk factors, diseases, and mortality [10–14], there is convincing evidence to recommend VPA in adults. Similarly, a systematic literature review supports the favorable influence of VPA (and MVPA) over LPA and MPA for fatness, aerobic fitness, and cardio-metabolic health outcomes in children [2]. Yet, interpretation and comparison of findings regarding possible PA intensity-specific influences across studies are hampered by great variability in accelerometer cut points used [2, 15], which leads to the capturing of somewhat different PA intensities. Thus, which activities and intensities that are captured in specific intensity zones differ among studies. This challenge can be solved by analyzing the intensity spectrum as a whole, irrespective of pre-defined cut points and selected PA intensity ranges.

Accelerometry provides a spectrum of PA intensity variables, ranging from zero to an upper threshold limited by the filtering option. However, the analysis of these raw intensity profiles represents a major statistical challenge due to the strong multi-collinearity between the variables. Common statistical methods, that is, ordinary least squares regression, cannot handle highly correlated variables. The traditional practice of limiting the number of exposure variables to a few variables (e.g., MVPA and SED) represents a possible solution to managing multi-collinear accelerometer variables, but this simplification comes at the cost of a potential large loss of information. Thus, we need novel statistical methods to overcome these challenges [16]. Although this need has stimulated the development of different analytic approaches in the field [17, 18], to the best of our knowledge, associations between PA and metabolic health have not been explored using multivariate pattern analysis. Multivariate pattern analysis is widely applied in pharmaceutical [19] and metabolomics studies [20], in addition to other fields of biomedical research, such as in treatment and diagnosis of diseases [21], with the objective of revealing patterns and important biomarkers among hundreds or even thousands of highly interrelated variables. Thus, as previously called for [2, 8], this statistical tool can treat accelerometer variables as a spectrum of intensities and thus provide detailed and greatly improved knowledge of multivariate associations – the signature – of PA related to metabolic health.

By using the whole PA intensity spectrum, the aim of the present study was to determine the intensity pattern that was associated with metabolic health in children. Thus, by means of multivariate pattern analysis, we sought to uncover the multivariate PA signature associated with child metabolic health including both single risk factors and a composite score as outcome measures.

## Methods

### Participants

The present study is a cross-sectional analysis using baseline data obtained from fifth-grade children in the Active Smarter Kids (ASK) cluster-randomized controlled trial, conducted in Norway during 2014–2015 [22, 23]. Sixty schools, encompassing 1202 fifth-grade children, fulfilled the inclusion criteria, and agreed to participate. This sample represented 86.2% of the population of 10-year-olds in the county, and 95.2% of those eligible for recruitment. Later, three schools encompassing a total of 27 fifth-grade children declined to participate. Thus, 1145 (97.4%) of 1175 available children from 57 schools agreed to participate in the study. Of these children, 841 (73.4%) children provided valid data for all variables relevant to the present analysis, and were included in the study.

Our procedures and methods conform to ethical guidelines defined by the World Medical Association’s Declaration of Helsinki and its subsequent revisions. The South-East Regional Committee for Medical Research Ethics in Norway approved the study protocol. We obtained written informed consent from each child’s parents or legal guardian and from the responsible school authorities prior to all testing. The study is registered in Clinicaltrials.gov with identification number: NCT02132494.

### Procedures

We have previously published a detailed description of the study [22], and therefore provide only a brief overview of the relevant procedures herein.

### Physical activity

PA was measured using the ActiGraph GT3X+ accelerometer (Pensacola, FL, USA) [24]. Participants were instructed to wear the accelerometer at the waist at all times over seven consecutive days, except during water activities (swimming, showering) or while sleeping. Units were initialized at a sampling rate of 30 Hz. Files were analyzed at 10-s epochs using the KineSoft analytical software version 3.3.80 (KineSoft, Loughborough, UK). Data were restricted to hours 06:00 to 23:59. In all analyses, consecutive periods of ≥60 min of zero counts were defined as non-wear time [15]. We applied wear time requirements of ≥8 h/day and ≥ 4 days/week to constitute a valid measurement.

We created 16 PA variables of total time (min/day) to capture movement in narrow intensity intervals throughout the spectrum; 0–99, 100–249, 250–499, 500–999, 1000–1499, 1500–1999, 2000–2499, 2500–2999, 3000–3499, 3500–3999, 4000–4499, 4500–4999, 5000–5999, 6000–6999, 7000–7999, and ≥ 8000 counts per minute (cpm). Sensitivity analyses were conducted to determine the influence of choosing previously suggested lower (0–49 cpm) or higher (0–149 cpm; 0–249 cpm) SED cut points [25, 26]. For the purpose of reporting descriptive statistics, we used the Evenson cut points of 0–99, 100–2295, 2296–4011, ≥ 4012, and ≥ 2296 cpm for SED, LPA, MPA, VPA, and MVPA [27, 28], respectively. We also reported achievement of the guideline PA level (mean of ≥60 min MVPA/day).

### Metabolic health measures

Aerobic fitness was measured with the Andersen intermittent running test, which has demonstrated acceptable reliability and validity in 10-year-old children [29]. Children ran as long as possible in a to-and-fro movement on a 20-m track, touching the floor with a hand each time they turned, with 15-s work periods and 15-s breaks, for a total duration of 10 min. The distance (meters) covered was used as the outcome. Body mass was measured using an electronic scale (Seca 899, SECA GmbH, Hamburg, Germany) with children wearing light clothing. Height was measured using a portable Seca 217 (SECA GmbH, Hamburg, Germany). Body mass index (BMI) (kg ·m^− 2^) was calculated and BMI status classified according to Cole et al. [30]. Waist circumference was measured with a Seca 201 (SECA GmbH, Hamburg, Germany) ergonomic circumference measuring tape two cm over the level of the umbilicus. Systolic (SBP) and diastolic blood pressures were measured using the Omron HBP-1300 automated blood pressure monitor (Omron Healthcare, Inc., Vernon Hills, IL, US). Children rested quietly for 10 min in a sitting position with no distractions before blood pressures was measured four times; we used the mean of the last three measurements for analyses. Serum blood samples were collected from the children’s antecubital vein between 08:00 and 10:00 in the morning after an overnight fast. All blood samples were analyzed for total cholesterol (TC), triglyceride (TG), high-density lipoprotein cholesterol (HDL), glucose, and insulin at the accredited Endocrine Laboratory of the VU Medical Center (VUmc; Amsterdam, the Netherlands). Low-density lipoprotein cholesterol (LDL) was estimated using the Friedewald formula [31]. We calculated the TC:HDL ratio and homeostasis model assessment (HOMA) (glucose (mmol/L) * insulin (pmol/L) / 22.5) [32].

We calculated a composite score as the mean of six variables (SBP, TG, TC:HDL ratio, HOMA, WC:height ratio, and the reversed Andersen test) by averaging standardized scores after adjustment for sex and age. A similar approach have been used previously [4]. The composite score was regarded the main outcome.

### Statistical analyses

Children’s characteristics were reported as frequencies, means, and standard deviations (SD). We tested for differences in characteristics between boys and girls, as well as between included and excluded children, using a linear mixed model to account for the clustering among schools. Models for PA and SED were adjusted for wear time. Bivariate associations among independent (PA) variables were described using Pearson’s correlation coefficient (r).

Associations between PA and metabolic health were determined using univariate statistics (Pearson’s r) and multivariate pattern analysis. Prior to performing these analyses, we performed ordinary least squares regression analyses with all metabolic health variables as dependent variables and obtained residuals from these models including age and sex as independent variables to adjust the outcomes for these variables and remove confounding. Adjustment for wear time did not change any finding, thus, unadjusted models are reported.

Partial least squares (PLS) regression analyses [33] were used to determine the multivariate PA signature of a suit of metabolic health measures (outcome variables), including all PA variables as explanatory variables. PLS regression decomposes the explanatory variables into orthogonal linear combinations (PLS components), while simultaneously it maximizes the covariance with the outcome variable. Thus, in contrast to ordinary least squares regression, PLS regression is able to handle completely collinear variables. Prior to PLS regression, all variables were centered and standardized to unit variance.

Monte Carlo resampling [34] with 100 repetitions was used to select the number of PLS components optimizing the predictive performance of the models. We simulated true prediction by repeatedly and randomly keeping 50% of the subjects as an external validation set when estimating the models. The predictive performance was calculated for an increasing number of components and the minimum median used as a criterion to determine the dimensions of the models. This ensures that the data are not over-fitted and thus guarantees statistical significance of the selected models. For each validated PLS regression model, a single predictive component was subsequently calculated by means of target projection [19, 35]. By this transformation all the predictive variance in the intensity spectrum related to the metabolic response variable is expressed in a single intensity vector. Selectivity ratios (SRs) were obtained as the ratio of this explained predictive variance to the residual variance for each PA intensity variable [36, 37]. The results are displayed in an SR plot indicating positive or negative associations with metabolic health. The sign of the SRs is determined from the corresponding loading on the predictive target projection component. The SR plots display quantitatively the PA variables according to their predictive and discriminatory importance for metabolic health. Confidence intervals were constructed around each SR and used to assess the significance of the SR for each PA variable. Addidionally, we reported the target projection loadings weighted by their SD (i.e., the covariance between PA variables and the metabolic health vector) to allow for a direct interpretation of the relative importance of change by a given duration (in minutes/day) among PA intensities. The procedure for obtaining the patterns is completely data-driven with no assumptions on variable distributions or degree of correlations between variables. Even complete collinearity between variables is handled by this analytic approach.

We compared the association patterns related to metabolic health for boys and girls by correlating the variable loadings from the separate multivariate models using Pearson’s r. The higher this correlation is, the higher is the similarity in association patterns for boys and girls and thus the more similar is the PA intensity pattern impacting metabolic health across gender.

Multivariate pattern analyses were performed by means of the commercial software Sirius version 11.0 (Pattern Recognition Systems AS, Bergen, Norway).

## Results

### Children’s characteristics

We included 841 children who provided valid data on all relevant variables (Table 1). The children (*n* = 841, 50% boys) included in the present analyses did not differ from the excluded children (*n* = 288, 57% boys) with respect to age (*p* ≥ .689) or anthropometry (*p* ≥ .166). Regarding indices of metabolic health, the included children performed better on the Andersen test (mean 898 (95% CI; 891–905) vs. 870 (856–884) meter, *p* < .001), and had lower fasting insulin concentrations (55.0 (52.9–57.0) vs. 64.5 (57.1–71.8) pmol/l, *p* = .001) and HOMA scores (1.71 (1.64–1.78) vs. 2.02 (1.78–2.27), *p* = .002) than the excluded children. Furthermore, the included children exhibited less SED time (490 (486–494) vs. 503 (493–512) min/day, p = .002) and spent more time in LPA (231 (229–234) vs. 226 (221–231) min/day, *p* = .031), MPA (44 (43–44) vs. 41 (39–43) min/day, *p* = .004), VPA (31 (30–32) vs. 27 (25–29) min/day, *p* = .010), and MVPA (74 (73–76) vs. 68 (64–72) min/day, *p* = .003) than the excluded children.

**Table 1:** Children’s baseline characteristics

	Overall (*n* = 841)	Boys (*n* = 424)	Girls (*n* = 417)	p between groups
Demography				
Age (years)	10.2 (0.3)	10.2 (0.3)	10.2 (0.3)	.803
Anthropometry				
Body mass (kg)	37.0 (8.1)	36.8 (7.8)	37.2 (8.3)	.641
Height (cm)	142.9 (6.7)	143.1 (6.7)	142.6 (6.8)	.197
BMI (kg/m^2^)	18.0 (3.0)	17.9 (2.9)	18.1 (3.1)	.218
Overweight and obese (%)	20.8	20.0	21.5	.583
Waist circumference (cm)	61.9 (7.5)	62.2 (7.3)	61.6 (7.7)	.169
Waist:height (ratio)	0.43 (0.05)	0.43 (0.05)	0.43 (0.05)	.322
Indices of metabolic health				
Andersen test (m)	898 (103)	925 (112)	871 (85)	< .001
Systolic blood pressure (mmHg)	105.2 (8.4)	105.3 (8.2)	105.2 (8.6)	.612
Diastolic blood pressure (mmHg)	57.7 (6.2)	57.4 (6.0)	58.1 (6.3)	.180
Total cholesterol (mmol/l)	4.46 (0.69)	4.46 (0.70)	4.46 (0.68)	.976
LDL cholesterol (mmol/l)	2.51 (0.64)	2.50 (0.65)	2.53 (0.62)	.570
HDL cholesterol (mmol/l)	1.59 (0.35)	1.63 (0.34)	1.55 (0.35)	.001
Total:HDL cholesterol (ratio)	2.91 (0.71)	2.82 (0.66)	2.99 (0.74)	.001
Triglyceride (mmol/l)	0.78 (0.38)	0.72 (0.31)	0.84 (0.42)	< .001
Glucose (mmol/l)	4.98 (0.32)	5.02 (0.31)	4.94 (0.33)	.001
Insulin (pmol/l)	55.0 (29.8)	48.9 (24.1)	61.1 (33.6)	< .001
HOMA (index)	1.71 (0.98)	1.54 (0.83)	1.89 (1.09)	< .001
Composite score (1SD)^a^	0.00 (1.00)	0.00 (0.93)	0.00 (1.07)	–
Physical activity^b^				
Wear time (min/day)	795 (57)	799 (59)	791 (54)	.038
Overall (cpm)	707 (271)	754 (296)	660 (235)	< .001
SED (min/day)	490 (60)	488 (63)	492 (57)	.018
LPA (min/day)	231 (38)	230 (40)	233 (37)	.022
MPA (min/day)	44 (13)	47 (13)	40 (11)	< .001
VPA (min/day)	31 (16)	34 (17)	27 (12)	< .001
MVPA (min/day)	74 (25)	81 (27)	67 (21)	< .001
Guideline amount (%)^c^	69	77	61	< .001

*BMI* body mass index, *LDL* low-density lipoprotein, *HDL* high-density lipoprotein, *HOMA* homeostasis model assessment

^a^ The composite score includes waist circumference, systolic blood pressure, total:HDL ratio, triglycerides, HOMA, and the Andersen test

^b^ Intensity-specific PA is calculated using the Evenson cut points [27]

^c^ Children achieving a mean of ≥60 min of MVPA per day

### Univariate statistics

Regarding inter-relationships among PA variables, time spent in the 0–99 cpm intensity interval correlated negatively with all other variables; the strongest correlations were found with time spent in intensity intervals from 1500 to 2999 cpm (*r* = − 0.51–-0.52). While all variables but PA in the 0–99 cpm intensity interval correlated strongly with the most proximal variables (*r* ≥ 0.91), correlations weakened gradually for more distal variables, but were in general positive.

Table 2 shows associations for each PA intensity interval variable with metabolic health, adjusted for age and sex, but not mutually adjusted for each other. A similar pattern emerged for all variables but SBP. Statistically significant unfavorable associations was seen for the 0–99 cpm intensity interval (SED) with the composite score, WC:height, TG, HOMA, and the Andersen test. No significant associations were seen for variables in the LPA range (≈100–2000 cpm). Associations were favorable and gradually stronger as the intensity increased in the MPA and VPA range.

**Table 2:** Correlations (Pearson’s r) for PA intensity intervals with metabolic risk, adjusted for age and sex

Variable (cpm)	Composite score	SBP	WC:height	TG	TC:HDL	HOMA	Andersen test
0–99	.09	−.05	.09	.09	.03	.10	−.08
100–249	.01	.01	−.01	.04	.00	.04	.02
250–499	.03	.02	.03	.04	.02	.03	.02
500–999	.03	.06	.04	.01	.02	.00	.03
1000–1499	.00	.09	.02	−.02	.00	−.03	.07
1500–1999	−.02	.10	.01	−.04	−.01	−.05	.11
2000–2499	−.05	.10	.00	−.06	−.03	−.06	.14
2500–2999	−.11	.07	−.04	−.10	−.08	−.10	.20
3000–3499	−.18	.04	−.10	−.12	−.11	−.13	.26
3500–3999	−.23	.02	−.16	−.13	−.14	−.17	.31
4000–4499	−.26	.03	−.22	−.13	−.16	−.19	.36
4500–4999	−.30	.02	−.26	−.15	−.17	−.22	.39
5000–5999	−.34	.03	−.31	−.16	−.19	−.25	.44
6000–6999	−.35	.02	−.32	−.15	−.20	−.25	.44
7000–6999	−.29	−.01	−.28	−.11	−.16	−.23	.36
≥ 8000	−.11	−.01	−.05	−.08	−.07	−.11	.09

*SBP* systolic blood pressure, *WC height* waist circumference to height ratio, *TG* triglyceride, *TC HDL* total cholesterol to high-density lipoprotein cholesterol ratio, *HOMA* homeostasis model assessment; Associations ≤ − .07 and ≥ .07 are significant at *p* < .05 without adjustment for multiple comparisons

### Multivariate pattern analyses

**Fig. 1:**
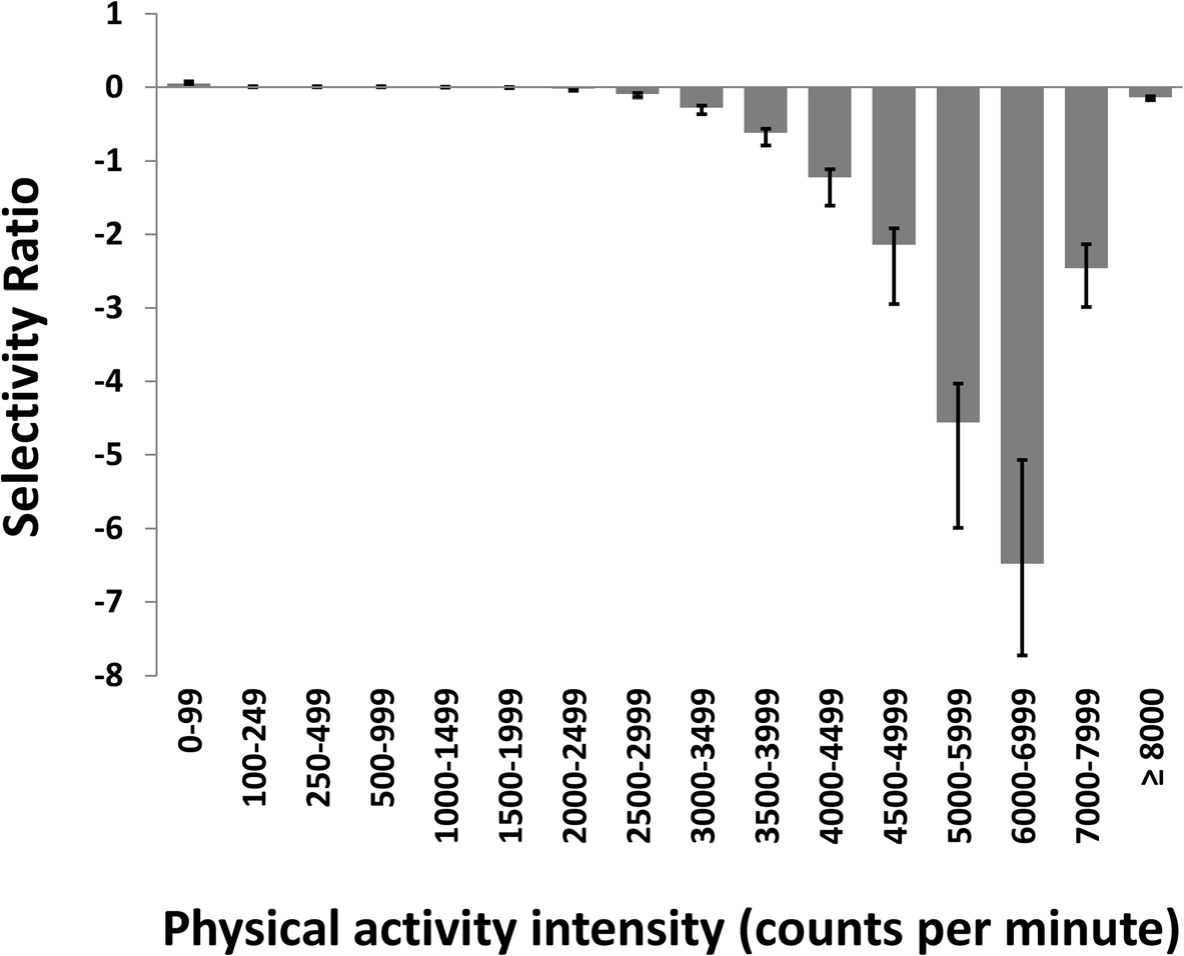
The multivariate PA signature associated with a composite metabolic health score in children displayed as a selectivity ratio (SR) plot. The PLS regression model includes 3 components, R^2^ = 13.3%, and is adjusted for age and sex. The SR for each variable is calculated as the ratio of explained to residual variance on the predictive (target projected) component. A negative bar implies that increased PA are associated with better metabolic health

**Fig. 2:**
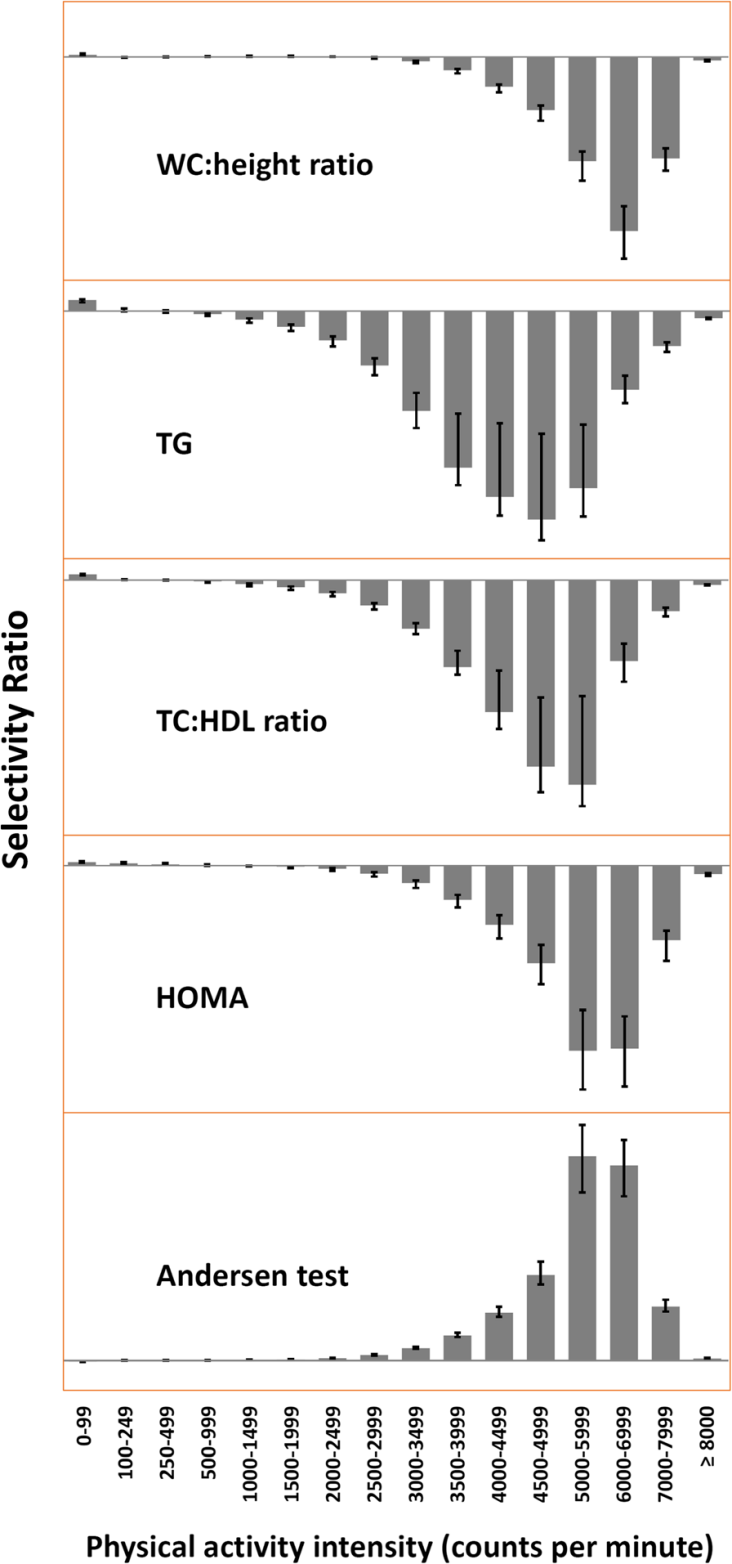
The multivariate PA signature associated different risk factors in children displayed as a selectivity ratio (SR) plot. The models (PLS regression) is adjusted for age and sex. WC:height ratio = waist circumference to height ratio (3 components, *R*^2^ = 13.6%); TG = triglyceride (1 component, *R*^2^ = 2.2%); TC:HDL ratio = total to high-density lipoprotein cholesterol ratio (1 component, *R*^2^ = 3.1%); HOMA = homeostasis model assessment (2 components, *R*^2^ = 6.6%); Andersen test (3 components, *R*^2^ = 21.0%). The SR for each variable is calculated as the ratio of explained to residual variance on the predictive (target projected) component. A negative bar implies that increased PA are associated with better metabolic health

**Fig. 3:**
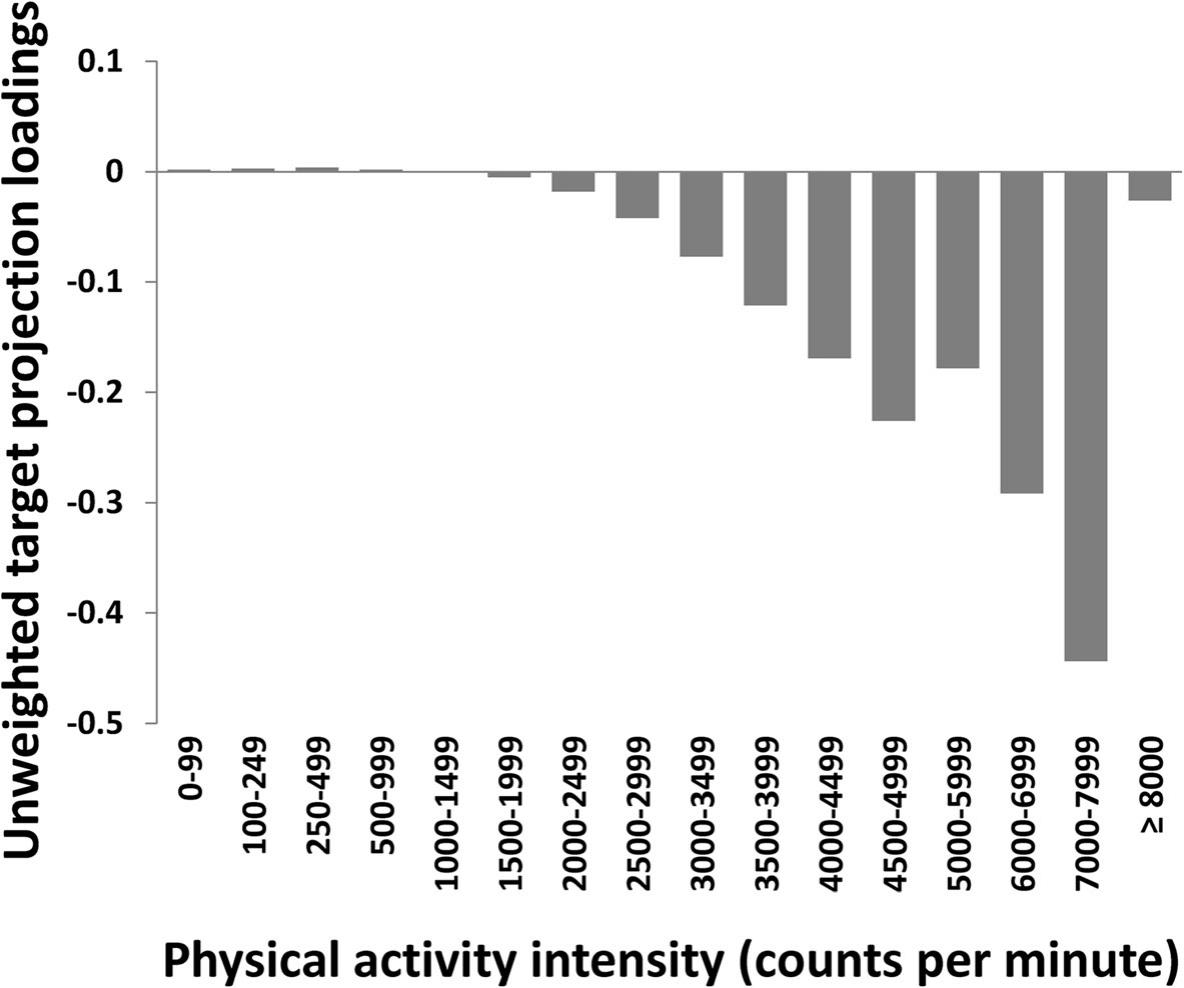
The unweighted target projection loadings for PA intensity intervals on the composite metabolic health vector in children. The figure shows the relative importance of the different PA intensity intervals for a given duration (minutes/day) of change. A negative bar implies that increased PA are associated with better metabolic health

Except for SBP, the association patterns for PA were rather similar for the composite score (Fig. 1) and all individual risk factors (Fig. 2). For SBP, a predictive multivariate association pattern did not exist (result not shown as the model was not statistically significant). PA intensity intervals between 5000 and 6999 cpm were most strongly related to metabolic health, while time spent in intensities below 3000 cpm was not related to metabolic health. The relative importance of a given duration (minutes/day) of each PA intensity for metabolic health is shown in Fig. 3. Partially in contrast to the patterns shown in Figs. 1 and 2, associations increased gradually from 2000 to 7999 cpm.

The association patterns in the multivariate pattern analyses were similar to those in the univariate correlation analyses (Table 2), but the unadjusted statistically significant associations with SED (0–99 cpm) for the composite score, WC:height, TG, HOMA, and the Andersen test were completely attenuated in the multivariate models (Fig. 1). The association patterns were similar for boys (3 PLS components, R^2^ = 16.3%) and girls (3 PLS components, R^2^ = 16.0%) (r for pattern of variable loading for boys and girls = 0.97, *p* < .001).

Sensitivity analyses revealed the results using SED cut points of < 50 (SR-ratio = 0.033), < 100 (SR-ratio = 0.033), < 150 (SR-ratio = 0.034), or < 250 cpm (SR-ratio = 0.034) were similar.

## Discussion

To handle a high number of strongly correlated intensity variables from accelerometry, we investigated the multivariate PA signature associated with metabolic health in children by means of multivariate pattern analyses. This novel approach shows for the first time how the whole intensity spectrum of PA associates to metabolic health in childhood. Our results clearly indicate strongest associations with metabolic health for VPA, weaker associations for MPA, and no associations for SED and LPA.

Because our analyses were restricted to cross-sectional associations, we could not infer causality from our findings. However, guidelines for PA are mainly based on population studies of free-living PA, because recommendations are related to *total* PA. Experimental studies can be used to establish causal relationships, but they per definition investigate effects of PA *in addition* to everyday living activities. Moreover, it would, in practice, be very challenging to conduct an experimental study informing the field like the present paper, due to the requirement of a large number of groups and participants, who would need very detailed and well-controlled exercise prescription and supervision. To this end, although we acknowledge the cross-sectional nature of the present study and the limitations it holds for drawing causal inferences, we argue the results presented are important for informing children’s guidelines for PA. Longitudinal data are needed to determine the predictive performance of the association pattern found, and whether association patterns with metabolic health differ in older children. Yet, besides weaker associations in longitudinal studies than in cross-sectional studies in general [2, 3], the favorable influence of PA of moderate to vigorous intensity on children’s metabolic health are evident irrespective of study design [1–4, 9, 38, 39].

Consistent with previous studies and recommendations [1–3, 9], our findings support that children should spend time in MVPA, or possibly just VPA, to improve their metabolic health. Although weak associations were found for intensities in the moderate range (see later for a discussion of cut points) [28], the association pattern was clearly dominated by intensities in the vigorous range. Derived from Fig. 3, it can be shown that the relative importance for metabolic health was 5 times greater for 1 min spent in typical vigorous intensities (mean of 5000–7999 cpm) compared to 1 min spent in typical moderate intensities (mean of 2500–3500 cpm). Thus, despite the clear dominance of the vigorous intensities, our analysis suggest a greater amount of MPA can substitute a lower amount of VPA. Similar to the findings from the systematic review by Cliff et al. [9], we found that SED was positively associated with adiposity and insulin sensitivity, and additionally with TG, and negatively associated with aerobic fitness, without adjustment for PA. When adjusted for PA in the multivariate model, the associations with SED vanished. Similarly, and consistent with the findings from the systematic review by Poitras et al. [2], PA intensities in the LPA range did not relate to any outcome in the present study. Yet, LPA and MPA were investigated in relatively few previous studies; thus, Poitras et al. [2] and others [8] called for inclusion of the whole intensity spectrum in future studies. Importantly, inclusion of the whole intensity spectrum also partly solve the challenge of residual confounding in accelerometer measurements, as it use much more of the available information from the accelerometer measurement [8]. Commonly applied statistical approaches do not allow for conducting these analyses. Given the dependency among the PA variables, our statistical approach is superior to the standard ordinary least squares approach because it can handle large collinear data sets [19, 33]. Thus, the multivariate pattern analysis applied in the present study enables us to determine the multivariate PA signature across the intensity spectrum with individual risk factors and a composite score of metabolic health.

In accordance with the current definition of SED [5], we agree SED (sitting or reclining) could be viewed as a behavior different from physical inactivity. Sitting is a separate construct of potential interest for public health surveillance and intervention, not just “physical inactivity by another name” [8]. Weak to moderate inter-correlations between SED and PA in both children and adults may support this view [40, 41], but this evidence is mainly based on subjective measures of SED and PA. Nevertheless, except for the posture of SED being sitting or reclined, it is indeed placed at the lower end of the intensity or energy expenditure spectrum (< 1.5 METs). Our findings clearly suggest – when analyzing the intensity spectrum as a whole – that SED is not important for children’s metabolic health. These findings illustrate the importance of sufficient control for confounding [8], as statistically significant associations with metabolic health were found using univariate statistics not controlling for PA, whereas associations for SED was completely attenuated when controlling for the full PA spectrum. This is an important finding for future research and efforts in the field, contrasting possible interpretations for other analytic approaches and designs. Although results from isotemporal substitution models [42] and experimental studies [43] suggest removal of SED is favorable for health, and thus might be interpreted to show the detrimental effect of SED per se, displacing SED inevitably means introducing PA [8]. Therefore, such studies cannot separate the influence of SED and PA. We argue, despite the cross-sectional nature of the present study, that our findings is a breakthrough relating to the call for solving the collinearity of PA data. Thus, it has important implications for future understanding and methodology in the field.

### Strengths and limitations

In addition to simultaneously modeling the whole intensity spectrum of PA and SED, a strength of the present study is the use of intensity ranges without respect to specific accelerometer cut points to define time spent in the SED, LPA, MPA, and VPA ranges. Because cut points vary considerably between studies [15], they hamper the interpretation of results regarding the different PA intensities’ importance for health. If, for example, we consider two influential studies in the field; Andersen et al. [4] defined MVPA above 2000 cpm and Ekelund et al. [3] defined MVPA above 3000 cpm. We argue a priori defined cut points might easily confuse findings and comparability among studies, when the same activities and intensities are captured by different intensity intervals across studies, or alternatively, different activities and intensities are captured by similar intensity intervals across studies. Thus, we believe using the whole intensity spectrum provide a much more nuanced picture of the associations between PA and metabolic health. Our findings suggest that in future studies there should be an increased focus on intensities in the upper range of MPA and VPA. Although this field is confused by inconsistencies [15], derived from a cross-validation, Trost et al. [28] suggests that moderate and vigorous PA intensities are best classified according to the Evenson et al. [27] (MPA ≥ 2296 and VPA ≥ 4012 cpm) and Freedson et al. [44] (MPA ≥ 2220 and VPA ≥ 4136 cpm standardized to a 12-year old) cut points. Our findings suggest that accumulated time > 4000–6000 cpm could be an important target for future analyses, if not using the whole intensity spectrum, and thus support the use of these VPA cut points as useful related to metabolic health. Limitations of the present study is the narrow age range of the children and a moderate sample size, but the similar association patterns in boys and girls indicate stability of our findings. The high response rate and the population-based sample are important strengths, but the generalizability should be interpreted with the differences between the children included and excluded from the analysis in mind. Future studies should attempt to replicate our findings using a similar analytic approach applied to larger data sets. Moreover, accelerometers do not provide a perfect measure of true SED time or very high PA intensities. Yet, agreement with direct observation of SED and activPAL is good on a group level, despite substantial variation at an individual level [25, 26]. Thus, our findings should be interpreted with regard to limited classification accuracy of posture allocation in mind. Additionally, the ActiGraph SED cut points applied in previous studies varies largely [15]. However, a sensitivity analysis of previously validated SED cut points in children [25, 26], revealed similar findings for cut points in the < 50–250 cpm range. Finally, the attenuated associations for the highest PA intensities (≥ 7000 cpm) could possibly be a spurious finding because of underestimation of these activities by the accelerometer, as it is well-known that ActiGraph counts levels-off for running [45, 46]. The accelerometer has a frequency filter (0.25–2.5 Hz) [24], which reduces the signal of high intensities to avoid noise, but it also reduces counts in the physiological range, possibly attenuating the relationship between high intensities and metabolic health.

## Conclusion

This study breaks new ground by using multivariate pattern analysis to investigate the PA signature of childhood metabolic health, including the whole spectrum of PA intensities. Our main conclusions are that the strongest associations with metabolic health exist for VPA, while there were weaker associations for MPA, and no associations for SED and LPA. Our findings suggest that future studies, recommendations and interventions should increase their focus on children’s time spent in VPA to capture the herein proposed strongest PA markers of childhood metabolic health. We further recommend that studies adapt the present multivariate analytic approach to develop the field of PA epidemiology.

## Abbreviations

CPM: Counts per minute
HDL: High-density lipoprotein cholesterol
HOMA: Homeostasis model assessment
LDL: Low-density lipoprotein cholesterol
LPA: Light physical activity
MPA: Moderate physical activity
MVPA: Moderate-to-vigorous physical activity
PA: Physical activity
PLS: Partial least squares
SBP: Systolic blood pressure
SED: Sedentary time
SR: Selectivity ratio
TC: Total cholesterol
TG: Triglyceride
VPA: Vigorous physical activity
WC: Waist circumference

## References

[1] Janssen I, LeBlanc AG. Systematic review of the health benefits of physical activity and fitness in school-aged children and youth. Int J Behav Nutr Phys Act. 2010;7 10.1186/1479-5868-7-40. 10.1186/1479-5868-7-40 PMC2885312 20459784

[2] Poitras VJ, Gray CE, Borghese MM, Carson V, Chaput JP, Janssen I (2016). Systematic review of the relationships between objectively measured physical activity and health indicators in school-aged children and youth. Appl Physiol Nutr Metab..

[3] Ekelund U, Luan JA, Sherar LB, Esliger DW, Griew P, Cooper A (2012). Moderate to vigorous physical activity and sedentary time and cardiometabolic risk factors in children and adolescents. JAMA.

[4] Andersen LB, Harro M, Sardinha LB, Froberg K, Ekelund U, Brage S (2006). Physical activity and clustered cardiovascular risk in children: a cross-sectional study (the European youth heart study). Lancet.

[5] Barnes J, Behrens TK, Benden ME, Biddle S, Bond D, Brassard P (2012). Letter to the editor: standardized use of the terms “sedentary” and “sedentary behaviours”. Appl Physiol Nutr Metab..

[6] Saunders TJ, Chaput JP, Tremblay MS (2014). Sedentary behaviour as an emerging risk factor for cardiometabolic diseases in children and youth. Can J Diabetes.

[7] Altenburg TM, Chinapaw MJM (2015). Bouts and breaks in children’s sedentary time: currently used operational definitions and recommendations for future research. Prev Med.

[8] van der Ploeg HP, Hillsdon M (2017). Is sedentary behaviour just physical inactivity by another name?. Int J Behav Nutr Phys Act.

[9] Cliff DP, Hesketh KD, Vella SA, Hinkley T, Tsiros MD, Ridgers ND (2016). Objectively measured sedentary behaviour and health and development in children and adolescents: systematic review and meta-analysis. Obes Rev.

[10] Kodama S, Saito K, Tanaka S, Maki M, Yachi Y, Asumi M (2009). Cardiorespiratory fitness as a quantitative predictor of all-cause mortality and cardiovascular events in healthy men and women a meta-analysis. JAMA.

[11] Swain DP, Franklin BA (2006). Comparison of cardioprotective benefits of vigorous versus moderate intensity aerobic exercise. Am J Cardiol.

[12] Milanovic Z, Sporis G, Weston M (2015). Effectiveness of high-intensity interval training (HIT) and continuous endurance training for VO_2max_ improvements: a systematic review and meta-analysis of controlled trials. Sports Med.

[13] Gebel K, Ding D, Chey T, Stamatakis E, Brown WJ, Bauman AE (2015). Effect of moderate to vigorous physical activity on all-cause mortality in middle-aged and older australians. JAMA Intern Med.

[14] Lavie CJ, Lee DC, Sui XM, Arena R, O'Keefe JH, Church TS (2015). Effects of running on chronic diseases and cardiovascular and all-cause mortality. Mayo Clin Proc.

[15] Cain KL, Sallis JF, Conway TL, Van Dyck D, Calhoon L (2013). Using accelerometers in youth physical activity studies: a review of methods. J Phys Act Health.

[16] Saunders TJ, Gray CE, Poitras VJ, Chaput JP, Janssen I, Katzmarzyk PT (2016). Combinations of physical activity, sedentary behaviour and sleep: relationships with health indicators in school-aged children and youth. Appl Physiol Nutr Metab.

[17] Chastin SFM, Palarea-Albaladejo J, Dontje ML, Skelton DA. Combined effects of time spent in physical activity, sedentary behaviors and sleep on obesity and cardio-metabolic health markers: A novel compositional data analysis approach. PLoS One. 2015;10(10) 10.1371/journal.pone.0139984. 10.1371/journal.pone.0139984 PMC4604082 26461112

[18] Mekary RA, Willett WC, Hu FB, Ding EL (2009). Isotemporal substitution paradigm for physical activity epidemiology and weight change. Am J Epidemiol.

[19] Rajalahti T, Kvalheim OM (2011). Multivariate data analysis in pharmaceutics: a tutorial review. Int J Pharm.

[20] Madsen R, Lundstedt T, Trygg J (2010). Chemometrics in metabolomics - a review in human disease diagnosis. Anal Chim Acta.

[21] Rajalahti T, Kroksveen AC, Arneberg R, Berven FS, Vedeler CA, Myhr K-M (2010). A multivariate approach to reveal biomarker signatures for disease classification: application to mass spectral profiles of cerebrospinal fluid from patients with multiple sclerosis. J Proteome Res.

[22] Resaland GK, Moe VF, Aadland E, Steene-Johannessen J, Glosvik Ø, Andersen JR (2015). Active Smarter Kids (ASK): Rationale and design of a cluster-randomized controlled trial investigating the effects of daily physical activity on children's academic performance and risk factors for non-communicable diseases. BMC Public Health.

[23] Resaland GK, Aadland E, Moe VF, Aadland KN, Skrede T, Stavnsbo M (2016). Effects of physical activity on schoolchildren’s academic performance: the active smarter kids (ASK) cluster-randomized controlled trial. Prev Med.

[24] John D, Freedson P (2012). ActiGraph and Actical physical activity monitors: a peek under the hood. Med Sci Sports Exerc.

[25] Ridgers ND, Salmon J, Ridley K, O'Connell E, Arundell L, Timperio A (2012). Agreement between activPAL and ActiGraph for assessing children's sedentary time. Int J Behav Nutr Phys Act.

[26] Ridley K, Ridgers ND, Salmon J (2016). Criterion validity of the activPAL (TM) and ActiGraph for assessing children's sitting and standing time in a school classroom setting. Int J Behav Nutr Phys Act.

[27] Evenson KR, Catellier DJ, Gill K, Ondrak KS, McMurray RG (2008). Calibration of two objective measures of physical activity for children. J Sports Sci.

[28] Trost SG, Loprinzi PD, Moore R, Pfeiffer KA (2011). Comparison of accelerometer cut points for predicting activity intensity in youth. Med Sci Sports Exerc.

[29] Aadland E, Terum T, Mamen A, Andersen LB, Resaland GK (2014). The Andersen aerobic fitness test: reliability and validity in 10-year-old children. PLoS One.

[30] Cole TJ, Bellizzi MC, Flegal KM, Dietz WH (2000). Establishing a standard definition for child overweight and obesity worldwide: International survey. BMJ.

[31] Friedewald WT, Levy RI, Fredrickson DS (1972). Estimation of the concentration of low-density lipoprotein cholesterol in plasma, without use of the preparative ultracentrifuge. Clin Chem.

[32] Matthews DR, Hosker JP, Rudenski AS, Naylor BA, Treacher DF, Turner RC (1985). Homeostasis model assessment: insulin resistance and β-cell function from fasting plasma glucose and insulin concentrations in man. Diabetologia.

[33] Wold S, Ruhe A, Wold H, Dunn WJ (1984). The collinearity problem in linear-regression - the partial least-squares (PLS) approach to generalized inverses. SIAM J Sci Comput.

[34] Kvalheim OM, Arneberg R, Grung B, Rajalahti T. Determination of optimum number of components in partial least squares regression from distributions of the root-mean-squared error obtained by Monte Carlo resampling. J Chemom. 2018; 10.1002/cem.2993.

[35] Kvalheim OM, Karstang TV (1989). Interpretation of latent-variable regression-models. Chemometr Intell Lab Syst.

[36] Rajalahti T, Arneberg R, Kroksveen AC, Berle M, Myhr KM, Kvalheim OM (2009). Discriminating variable test and selectivity ratio plot: quantitative tools for interpretation and variable (biomarker) selection in complex spectral or chromatographic profiles. Anal Chem.

[37] Rajalahti T, Arneberg R, Berven FS, Myhr KM, Ulvik RJ, Kvalheim OM (2009). Biomarker discovery in mass spectral profiles by means of selectivity ratio plot. Chemometr Intell Lab Syst..

[38] Stamatakis E, Coombs N, Tiling K, Mattocks C, Cooper A, Hardy LL (2015). Sedentary time in late childhood and cardiometabolic risk in adolescence. Pediatrics.

[39] Chinapaw M, Klakk H, Moller NC, Andersen LB, Altenburg T, Wedderkopp N. Total volume versus bouts: prospective relationship of physical activity and sendetary time with cardiometabolic risk in children. Int J Obes. 2018; 10.1038/s41366-018-0063-8. 10.1038/s41366-018-0063-8 29717272

[40] Pearson N, Braithwaite RE, Biddle SJH, van Sluijs EMF, Atkin AJ (2014). Associations between sedentary behaviour and physical activity in children and adolescents: a meta-analysis. Obes Rev.

[41] Mansoubi M, Pearson N, Biddle SJH, Clemes S (2014). The relationship between sedentary behaviour and physical activity in adults: a systematic review. Prev Med.

[42] Garcia-Hermoso A, Saavedra JM, Ramirez-Velez R, Ekelund U, del Pozo-Cruz B (2017). Reallocating sedentary time to moderate-to-vigorous physical activity but not to light-intensity physical activity is effective to reduce adiposity among youths: a systematic review and meta-analysis. Obes Rev.

[43] Benatti FB, Ried-Larsen M (2015). The effects of breaking up prolonged sitting time: a review of experimental studies. Med Sci Sports Exerc.

[44] Freedson P, Pober D, Janz KF (2005). Calibration of accelerometer output for children. Med Sci Sports Exerc.

[45] Brage S, Wedderkopp N, Franks PW, Andersen LB, Froberg K (2003). Reexamination of validity and reliability of the CSA monitor in walking and running. Med Sci Sports Exerc.

[46] John D, Miller R, Kozey-Keadle S, Caldwell G, Freedson P (2012). Biomechanical examination of the ‘plateau phenomenon’ in ActiGraph vertical activity counts. Physiol Meas.

